# Genome-wide demethylation and targeted remethylation during metamorphosis in the jewel wasp *Nasonia vitripennis*

**DOI:** 10.1186/s13072-025-00639-w

**Published:** 2025-12-04

**Authors:** C. L. Thomas, E. B. Mallon

**Affiliations:** Division of Genetics and Genome Biology, School of Biological and Biomedical Sciences, University of Leicester, University Road, Leicester, LE1 7RH UK

**Keywords:** DNA methylation, Insect development, Epigenetics, Transcriptional regulation, Metamorphosis, *Nasonia vitripennis*

## Abstract

DNA methylation plays a critical regulatory role during insect development, yet the underlying mechanisms remain poorly understood. Here, we provide a comprehensive profile of DNA methylation dynamics across the developmental stages of the parasitoid wasp *Nasonia vitripennis*, a key insect model with functional methylation machinery. Using whole-genome bisulfite sequencing, we identify stage-specific methylation levels, including substantial genome-wide demethylation during the embryonic-to-larval transition and remethylation during subsequent metamorphic stages. Differential methylation analyses reveal significant enrichment of developmentally relevant Gene Ontology terms, highlighting roles in gastrulation, embryogenesis, larval development, regionalisation and morphogenesis. Analysis of protein binding motifs at differentially methylated sites further suggests DNA methylation may directly modulate transcription factor activity, a regulatory mechanism previously underappreciated in insects methylomics. RNA sequencing reveals coordinated expression of methylation-associated enzymes, including high embryonic expression of the demethylase *tet* and the methylation reader *mbd*, consistent with methylation dynamics. Although the regulatory relationship between DNA methylation and gene expression is complex, we observed that methylation may contribute to developmental transitions by influencing transcription factor accessibility and chromatin state. Our results suggest that DNA methylation levels are dynamic across *Nasonia* metamorphosis, and may modulate transcription factor binding across development. These findings refine current models of epigenetic regulation in holometabolous insects and establish a *Nasonia vitripennis* methylome across metamorphosis for the first time.

**Supplementary Information:**

The online version contains supplementary material available at 10.1186/s13072-025-00639-w.

## Background

DNA methylation, a pivotal epigenetic modification involving the addition of a methyl group to cytosine, profoundly influences gene regulation. While extensively studied in mammals where it undergoes dynamic developmental reprogramming [1, 2], recent findings suggest that insects also exhibit stage-specific methylation dynamics, though these remain poorly characterised. Across insect species, interference of DNMT enzymes often leads to developmental arrest or reproductive disruption [3–7]. In the silk moth *Bombyx mori*, DNA methylation has recently been shown to regulate gene expression through recruitment of chromatin modifiers, notably the histone acetyltransferase Tip60, influencing transcriptional activation during early embryogenesis [8]. Even *Drosophila melanogaster*, which lacks conventional methylation machinery, exhibits transient methylation fluctuations [9], and methylation is a key modulator of metamorphosic processes [10], underscoring the importance of understanding insect-specific methylation mechanisms.

In mammals, DNA methylation primarily modulates gene expression by recruiting or inhibiting transcription-associated proteins, including methyl-CpG-binding domain proteins (MBDs), which attract chromatin remodelers and histone-modifying enzymes to methylated genomic regions [11]. However, in insects, the functional consequences of gene body methylation remain less clear, and the mechanistic link between DNA methylation and transcriptional outcomes, particularly across developmental transitions in holometabolous species, is poorly defined. Given that methylation may also directly modulate transcription factor binding [12], understanding how methylation might regulate insect development via multiple mechanisms remains a key open question.

*Nasonia vitripennis* was amongst the first insect species to show that disrupting DNA methylation levels resulted in developmental failure [3]. However, it has also been proposed that DNA methylation remains relatively stable between the embryo and adult stage [6, 13]. A growing number of studies are using *Nasonia* as an insect species to study DNA methylation, in part due to its short life cycle, compact genome, laboratory tractability, possession of both *dnmt1* (3 homologs) and *dnmt3* (1 homolog)[14]. Despite the frequency that *Nasonia* is used in insect methylation studies, it still remains unclear whether methylation is dynamically reprogrammed across its entire developmental trajectory, particularly during critical metamorphic transitions, and how such changes might influence gene expression.

Here, we profile the developmental DNA methylation landscape across the complete life cycle of *Nasonia vitripennis*, integrating differential methylation analyses, transcription factor motif predictions, and RNA sequencing to uncover how methylation may regulate gene expression during metamorphosis. By examining the expression of key methylation enzymes, we identify potential contributors to global methylation changes. These findings allow us to hypothesise potential drivers of epigenetic regulation during insect development and provide a framework for broader comparative studies of developmental methylation dynamics.

## Methods

### Sample collection

Wasps used in this study were of the species *Nasonia vitripennis* (Leicester strain) [15]. Stocks were maintained on 20% sucrose solution under controlled conditions: 25^o^C, 40% humidity, and a 12-hour light/dark cycle.

The study focused on female *Nasonia*. Parental wasps were collected within 24 "hours" of eclosion and placed in groups of three females and one male. These groups were provided with a “practice” host (placed in a wide-bore pipette tip pierced through a cotton bung) and filter paper soaked with 20% sucrose for 24 h to facilitate egg collection. After replacing the practice host with a fresh host, wasps were allowed three hours to oviposit. The host was then removed and incubated until sample collection. This protocol resulted in 83.3% female offspring ($n = 944$), consistent with previous studies (80–95%) [16, 17]. Developmental stage samples were collected as follows: embryos (7–10 h post-laying), larvae (69–72 h post-laying), prepupae (7 days post-laying), pupae (yellow-eye stage, 9 days post-laying), and adults (24 h post-eclosion). Adults and pupae were sexed by inspection.

### DNA extraction for whole genome bisulfite sequencing (WGBS)

DNA was extracted from 1,700 embryos, 30 larvae, 10 prepupae, 10 pupae, and 10 adults per replicate using the DNeasy Blood & Tissue Kit (Qiagen), with modifications to the standard protocol (see [18]). Samples were incubated with (Purelink 20 mg/ml) RNase A for 30 min, with the exception of RNA rich larva which were incubated for 90 min. Three biological replicates were collected per stage, except for embryos, where one replicate failed quality control. DNA quality was assessed using a NanoDrop 2000 spectrophotometer (Thermo Scientific), Qubit^TM^ dsDNA BR Assay Kit (Thermo Fisher Scientific), and 1% agarose gel electrophoresis. Samples were sent to BGI Genomics for bisulfite treatment, lambda spiking, adapter ligation, sequencing, and trimming.

### RNA extraction for RNA sequencing

RNA was extracted from 400 embryos, 30 larvae, 10 prepupae, 10 pupae, and 10 adults per replicate (three replicates per stage) using Tri-Reagent (Sigma Aldrich) with modifications for TURBO DNase^TM^ treatment and removal (see [18]). RNA concentration was measured using the Qubit^TM^ RNA BR Assay Kit (Thermo Fisher Scientific), and RNA integrity was verified on an Agilent 2100 Bioanalyzer using the RNA 6000 Nano Kit (Agilent). Sequencing and trimming were performed at BGI Genomics.

### Bioinformatics

#### DNA methylation

To provide an overview of methylated sites at each developmental stage, WGBS replicates for each developmental stage were merged to create a single FASTQ file per stage. These files were aligned to the Nvit_psr_1.1 genome [19] using Bismark [20]. As each sample contained a lambda spike, we could estimate the false-positive methylation rate of bisulphite conversion by also aligning samples to the lambda genome. PCR duplicates were removed using the Bismark deduplicate command, and methylation levels were extracted using the bismark_methylation_extractor command [20]. Destranding of methylation data was performed with the coverage2cytosine command to improve coverage [20]. To determine genomic locations which were methylated, a binomial test was conducted in R (version 4.3.1) for each genomic CpG site using the non-conversion rate (estimated from the alignment to the lambda genome) as the probability of success. CpGs with an FDR < 0.05 were considered to be methylated.

For differential methylation analyses, each replicate was processed individually as described above. Filtering was performed using the R package methylkit [21], retaining only CpGs with a minimum coverage of 10 reads. Principal component analysis (PCA) was performed with the PCASamples function in methylkit. 4 pairwise comparisons were conducted: embryo vs. larva, larva vs. prepupa, prepupa vs. pupa, and pupa vs. adult. Differentially methylated sites were determined using the calculateDiffMeth() function in methylkit [21] which uses a logistic regression. Differentially methylated CpGs were defined as those with a statistically significant difference (P < 0.0125, Bonferroni correction) and a methylation difference exceeding 10%.

Genomic features were assigned to methylated sites using a GFF file generated with AGAT [22]. Transcription factor binding sites were predicted using TFBSTools [23] with the JASPAR 2024 *Drosophila melanogaster* transcription factor database [24], retaining only matches with 100% homology. Enriched genomic motifs were identified using HOMER [25], with the-size parameter set to 100. Gene Ontology (GO) terms were retrieved from EggNOG-Mapper [26] and enrichment analysis was performed using the R package TopGO [27] using the elim algorithm. KEGG pathway analysis was performed using ClusterProfile [28] using KO terms generated from EggNOG-Mapper [26].

Whole genome bisulfite sequencing files are available on EBI under the accession number E-MTAB-14656.

#### RNA sequencing

Paired-end RNA reads were aligned to the Nvit_psr_1.1 genome [19] using the STAR two-pass alignment method (version 2.7.5c) [29]. Read counts were generated with HTSeq [30] and normalized using DESeq2 (version 1.32.0) [31]. Fragments per million mapped reads (FPM) were calculated using the fpm() function in DESeq2 and visualized with ggplot2 [32]. To calculate the difference in gene expression of methylation associated genes, we plotted a linear model lm(Counts $\sim$ Condition * Gene), with post-hoc pairwise comparisons being performed using estimated marginal means (emmeans) from the fitted model. Exon-level counts were obtained using DEXSeq [33]. Isoform abundances were generated using Kallisto [34]. RNA sequencing libraries are publicly available on EBI under the accession number E-MTAB-14657.

#### Modeling DNA methylation and gene expression

We used generalized linear models (GLMs) to analyze the effects of DNA methylation on gene expression. For models examining raw counts, we applied a quasi-Poisson distribution, while for models examining log-fold changes (LFC) between stages, we used Gaussian-distributed GLMs. The models were specified as follows:

1 $$\begin{aligned}&{\textbf {Gene Counts}} \sim {\textbf {Methylation Percentage}} \times {\textbf {Developmental Stage}}\end{aligned}$$

2 $$\begin{aligned}&{\textbf {Exon Counts}} \sim {\textbf {Methylation Percentage}} \times {\textbf {Developmental Stage}}\end{aligned}$$

3 $$\begin{aligned}&{\textbf {Gene Counts}} \sim {\textbf {Methylation Class}} \times {\textbf {Developmental Stage}}\end{aligned}$$

4 $$\begin{aligned}&{\textbf {LFC}} \sim {\textbf {No. Differentially Methylated Sites}} \times {\textbf {Comparison}}\end{aligned}$$

5 $$\begin{aligned}&{\textbf {LFC}} \sim {\textbf {Distance TSS}} \times {\textbf {Transcription Factor}} \times {\textbf {Comparison}}\end{aligned}$$

6 $$\begin{aligned}&{\textbf {LFC}} \sim {\textbf {Distance TSS}} \times {\textbf {Comparison}} \times {\textbf {No. Consecutive CpGs}} \end{aligned}$$

For model 3, genes were categorized into four methylation classes (high: 70–100%, medium: 30–70%, low: 0.5–30%, or unmethylated: <0.5%) following established thresholds [35].

For models using percentage methylation levels (1-2) and methylation class (3), regular RNA-seq counts were modeled; for models comparing developmental stages (4-6), log-fold changes in gene expression were used. In these models (4-6), comparison is the pairwise comparison in question (e.g. embryo vs. larva).

All scripts for annotation, methylation processing, and RNA analysis are available at: https://github.com/C-L-Thomas/Nasonia_Developmental_Methylation.

## Results

### Developmental stage methylation

The *Nasonia vitripennis* genome contains 16,515,404 destranded CpG sites, of which 14,903,835 to 15,084,779 had a coverage of at least 10 reads when combining all reads for each developmental stage. Methylated sites were defined as CpGs that passed a binomial test with the false-positive rate calculated using a lambda spike for each bisulfite-treated sample. The proportion of destranded methylated sites was relatively consistent across developmental stages: 182,740 in embryos (1.2% of CpGs), 170,115 in larvae (1.1%), 178,611 in prepupae (1.2%), 182,221 in pupae (1.2%), and 178,309 in adults (1.2%).

**Fig. 1:**
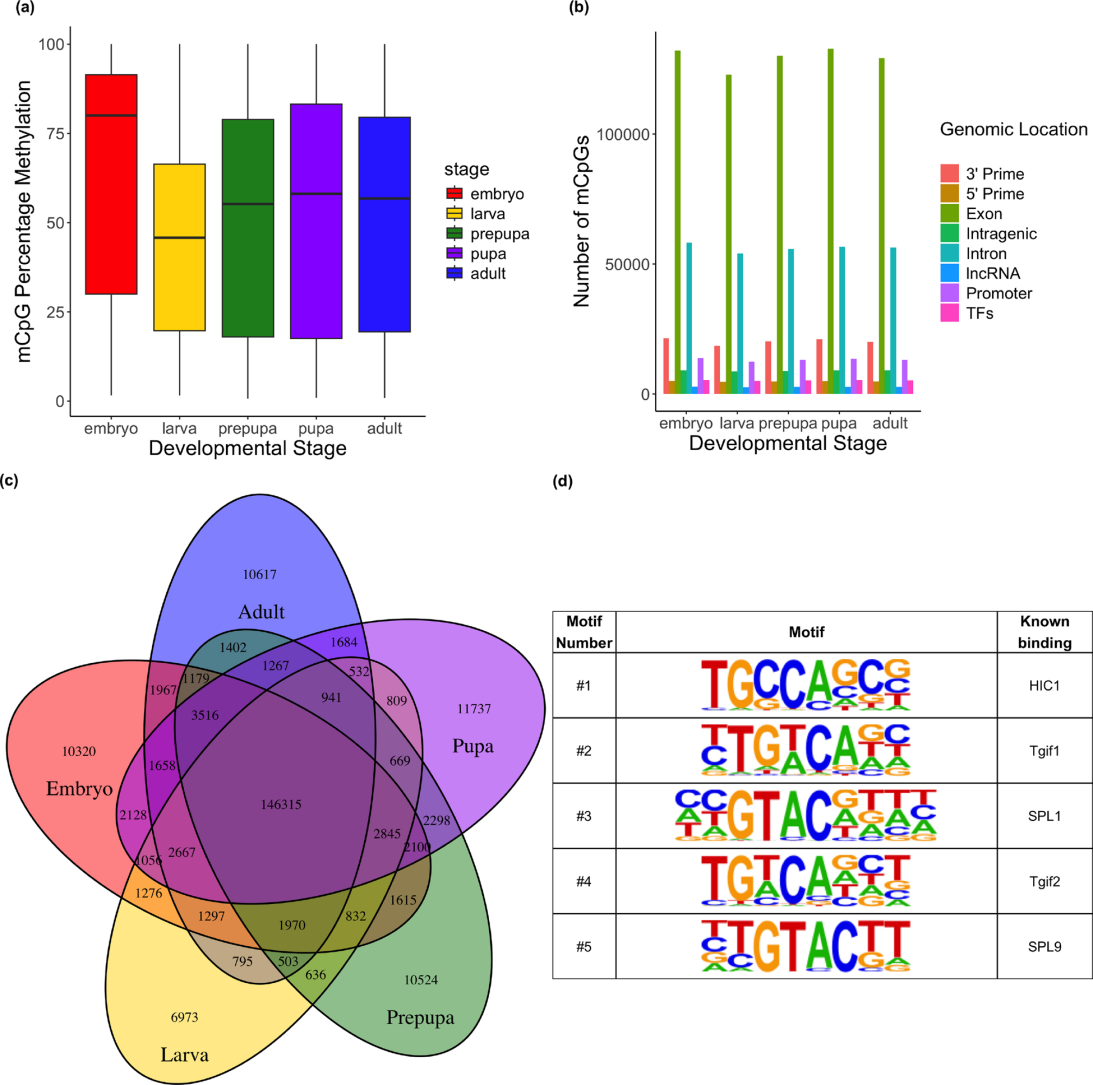
Developmental Stage Methylation. **A** Percentage methylation for CpGs that passed the binomial test (i.e., are methylated). **B** Genomic distribution of methylated sites for each developmental stage. **C** Venn diagram showing the number of CpGs methylated across all developmental stages. **D** Top five enriched motifs of CpGs methylated in all five developmental stages

The embryo exhibited the highest percentage of methylation (Figure 1A), and was followed by the lowest percentage of methylation in the larval stage. Methylation levels increased slightly in the prepupal stage and then stabilized through subsequent stages. The genomic distribution of methylated CpGs was consistent across all stages (Figure 1B), with the majority located within gene bodies, particularly in exons.

A total of 146,315 CpGs were methylated across all developmental stages (Figure 1C). GO term enrichment of the genes that these CpGs were located in revealed a mix of some stage specific processes (eg. gastrulation, and larval development), and core functions (eg. splicing, chromosome organization and ribosome biogenesis and organic substance metabolic process). We performed motif analysis on CpGs that were methylated across all developmental stages to identify sequence features associated with consistent methylation (Figure 1D). Amongst the top enriched known motifs we find enrichment of binding sites implicated core biological processes including splicing regulation (Splice donor and Splice acceptor) and chromatin structure (Znf263 and Zfx). Motif analysis of CpG-centered 6-mers revealed strong enrichment for T/A-rich flanks. The most enriched contexts were TACGTA (2.63-fold; $\log _{2}=+1.39$; $\textrm{OR}_{\textrm{H}}=2.65$; Fisher’s exact two-sided $p \approx 1.1\times 10^{-295}$, FDR < 0.0001) and ATCGTA (2.62-fold; $\log _{2}=+1.39$; $\textrm{OR}_{\textrm{H}}=2.64$; $p \approx 9.9\times 10^{-242}$, FDR < 0.0001).

Next, we performed GO and KEGG enrichment on the genes which were uniquely methylated in individual developmental stages (Figure 1C). The embryo revealed biological process terms for action potential, gastrulation, regionalization and embryonic morphogenesis, but revealed no KEGG enrichment. The larval stage produced GO terms for action potential, angiogenesis, regionalization, anatomical structure morphogenesis and epithelium development, whilst KEGG analysis found enrichment for axon guidance, hippo signaling pathway (fly), Rap1 signaling pathway and cytoskeleton in muscle cells. The prepupal stage produced GO terms for action potential, epithelial cell development, cell development cell differentiation and regulation of membrane potential, and KEGG analysis revealed enrichment for the insulin signaling pathway, hippo signaling pathway (fly), neurotrophin signaling pathway, and AMPK signaling pathway. The pupal stage produced GO terms for cell development, cell differentiation, vasculature development and epithelial cell differentiation, and produced a KEGG term for hippo signaling pathway (fly). Finally, the adult stage was enriched for GO terms associated with action potential, regionalization, epithelial cell development, cell differentiation, and regulation of membrane potential, but generated no KEGG pathways.

We performed motif enrichment analysis on the regions surrounding each stage’s unique CpGs to elucidate whether DNA methylation could regulate protein binding during development. In the embryo we found enrichment of binding sites that shared homology with Slp1, Pho, Sox10, Six4 and Vdr binding sites. For sites exclusively methylated in the larval stage, CpGs were located in an enriched motif that shared homology for Vnd binding. CpGs methylated exclusively in the prepupa were examined for motif enrichment, and we found binding sites that were homologous to NeuroD1, Aef1 and Odr-7 binding sites. CpGs uniquely methylated in the pupa showed motif enrichment for sites homologous to binding sites for Zfx, Nrf1, and Brk. Finally, in the adult, motifs were enriched for Ceh-60, Vvl, and Grh.

### Differential methylation

**Fig. 2:**
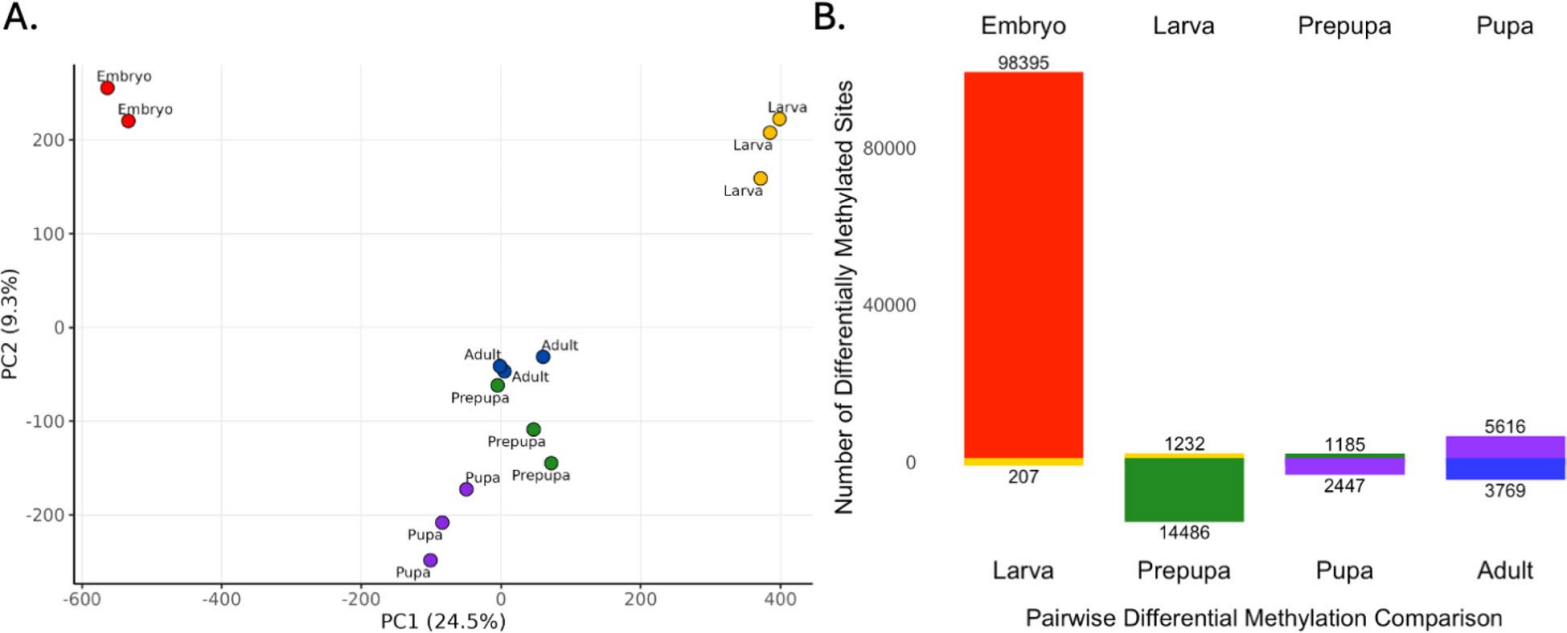
Differential Methylation Results. **A** PCA of individual whole-genome bisulfite sequencing samples. **B** Pairwise comparisons showing the number of differentially methylated CpGs between embryo (red) and larva (yellow), larva and prepupa (green), prepupa and pupa (purple), and pupa and adult (blue)

To identify differentially methylated CpGs, we performed pairwise analyses across the four developmental stage transitions (embryo to larva, larva to prepupa, prepupa to pupa and pupa to adult). At the CpG level, replicates from each stage clustered distinctly in a PCA (Figure 2A). The genomic distribution of differentially methylated CpGs (e.g., exon, intron, intragenic) mirrored the distribution of total methylation (Figure 1b and Supplementary Figure 2), indicating no specific preference for genomic locations. Whole-gene differential methylation levels are provided in Supplementary Information 2.

#### Embryonic to larval transition

The largest number of differentially methylated CpGs (98,602) occurred between the embryonic and larval stages, with 98,395 hypermethylated in embryos and 207 in larvae (Figure 2B). GO terms for genes these differentially methylated CpGs were located within were associated with gastrulation, larval development, chordate embryonic development, urogenital system development, regionalization, cell differentiation, and embryonic morphogenesis. Motif analysis of the regions that include these differentially methylated CpGs revealed sites similar to Pros, Tra2, Run and Optix binding sites.

#### Larval to prepupal transition

Between the larval and prepupal stages, 15,718 differentially methylated CpGs were identified, with the majority hypermethylated in prepupae (Figure 2B). GO term analysis of the genes that these CpGs were located in revealed enrichment for terms associated with larval development, anatomical structure formation involved in morphogenesis, regionalization, larval development, and cell differentiation. Motif enrichement of the regions surround these CpGs revealed binding sites homologous to those of Nfix, Sna, Tra2, Adf1, Zelda, and Hoxa9 binding sites.

#### Prepupal to pupal transition

In the prepupal to pupal transition, 3,632 CpGs were differentially methylated, with 2,447 hypermethylated in pupae and 1,185 in prepupae. GO term analysis of the genes these differentially methylated CpGs were located in included larval development, epithelial cell development, cell differentiation and signal transduction. Enriched motifs that these CpGs were located within shared homology with Dsx, Adf1, Pho and Kni binding sites.

#### Pupal to adult transition

Between the pupal and adult stages, 9,385 CpGs were differentially methylated, with 5,616 hypermethylated in pupae and 3,769 in adults. GO term enrichment of the genes that these CpGs were located in produced GO terms for regionalization, cell differentiation, vasculature development, cellular response to stimulus and signal transduction. Motif analysis of the region surrounding these differentially methylated sites identified binding sites homologous to those of Lsl-1, Pax-2, Cad, Mef2, and Ovo.

### DNA methylation enzyme genes’ expression during development

**Fig. 3:**
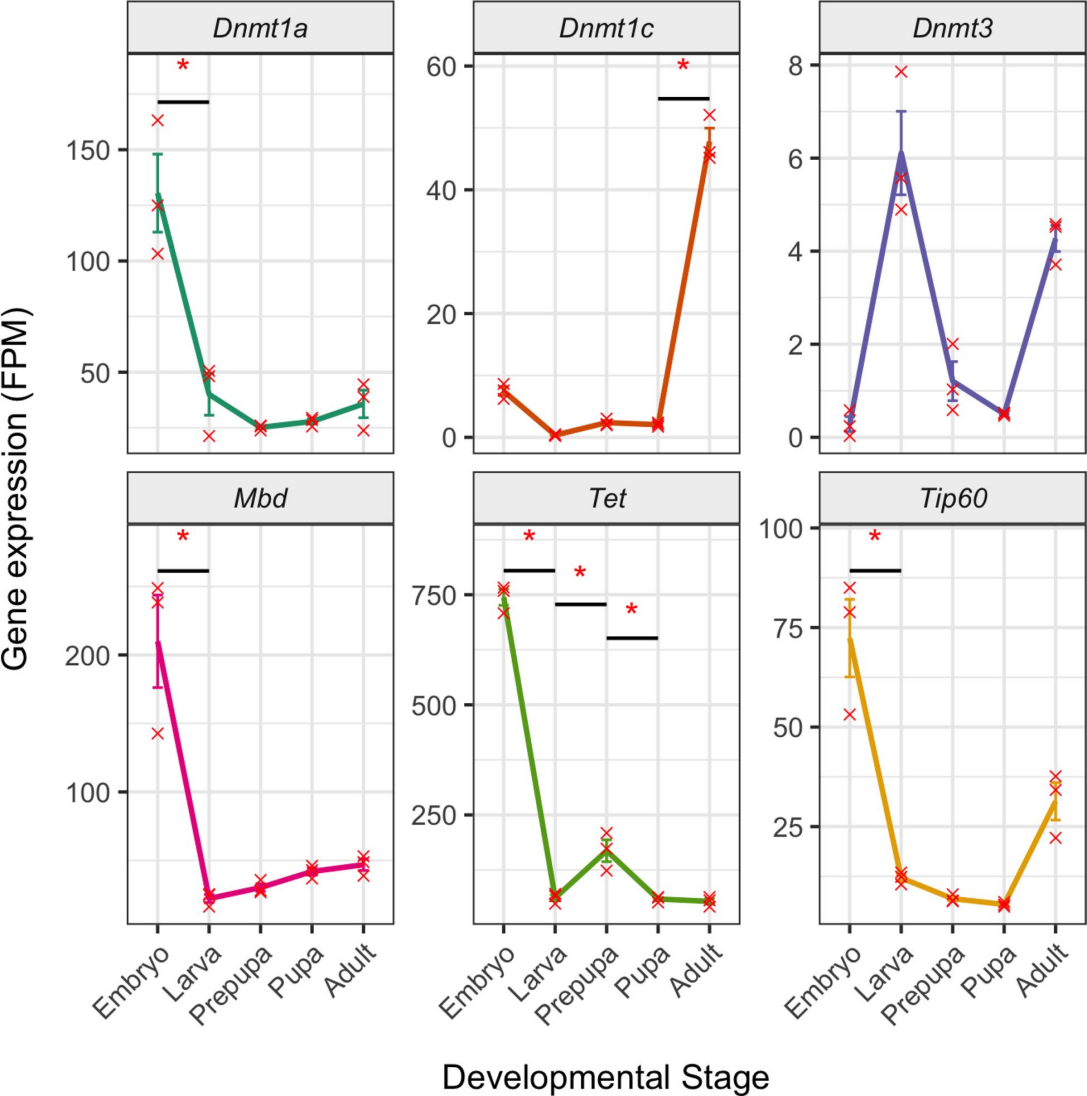
RNA sequencing fragments per million mapped (FPM) of genes associated with DNA methylation across developmental stages, fitted with standard error bars. Significant differences between stage are donated by an asterisk, and associated stats can be found in Supplementary Information 3

The demethylase enzyme *tet* showed highest expression during the embryonic stage (Figure 3), with a significant decrease in expression ($\beta = 682.712, \, SE = 13.60, \, t(60) = 50.045, \,$, *p* < .0001) that coincided with the largest demethylation event observed between the embryonic and larval stages (Figures 1A and 2B). There was also a signficant difference in *tet* expression between the larva and prepupal stage ($\beta = -107.015, \, SE = 13.6, \, t(60) = -7.845,$*p* < .0001) and the prepupal and pupal stage ($\beta = 109.532, \, SE = 13.6, \, t(60) = 8.029$, *p* < .0001). The expression of *dnmt1a* was also highest in the embryos, with a significant decrease in expression in the transition between the embryo and the larva ($\beta = 90.43, \, SE = 13.60, \, t(60) = 6.63$, *p* < .001) followed by stable expression through the remaining stages. *Dnmt1a* was highest expressed *dnmt* enzyme throughout development. Similarly, we see the highest expression of the DNA methylation gene expression linking proteins *mbd* and *tip60* in the embryo stage, with much lower expression levels in the remainder of metamorphosis Supplementary Information 3. Expression of the de novo methyltransferase *dnmt3* is at its highest in the larval stage, coinciding with the increase of methylation entering the prepupal stage (Figure 1A). Meanwhile, expression of the suspected ovary specific methyltransferase *dnmt1c* [18] peaks in the adult stage. No expression of *dnmt1b* was detected. Full results for these statistical tests can be found in Supplementary Information 3.

We also observed isoform usage, and found that an *mbd* isoform that contains a Hymenoptera specific domain where a CpG binding domain should be (NM_001171055.1) is more abundant in the adult, whilst the isoform which lacks this domain is more abundant in the embryo (NM_001171055.1) Supplementatry Figures (3). Additionally, one *tet* isoforms that contains a CXXC zinc finger domain have highest abundance in the embryo, whilst an isoform that does not (XM_008205120.3) has equally high abundance in the adult stage as the embryo (Supplementatry Figures 3).

### DNA methylation’s effect on gene expression

**Fig. 4:**
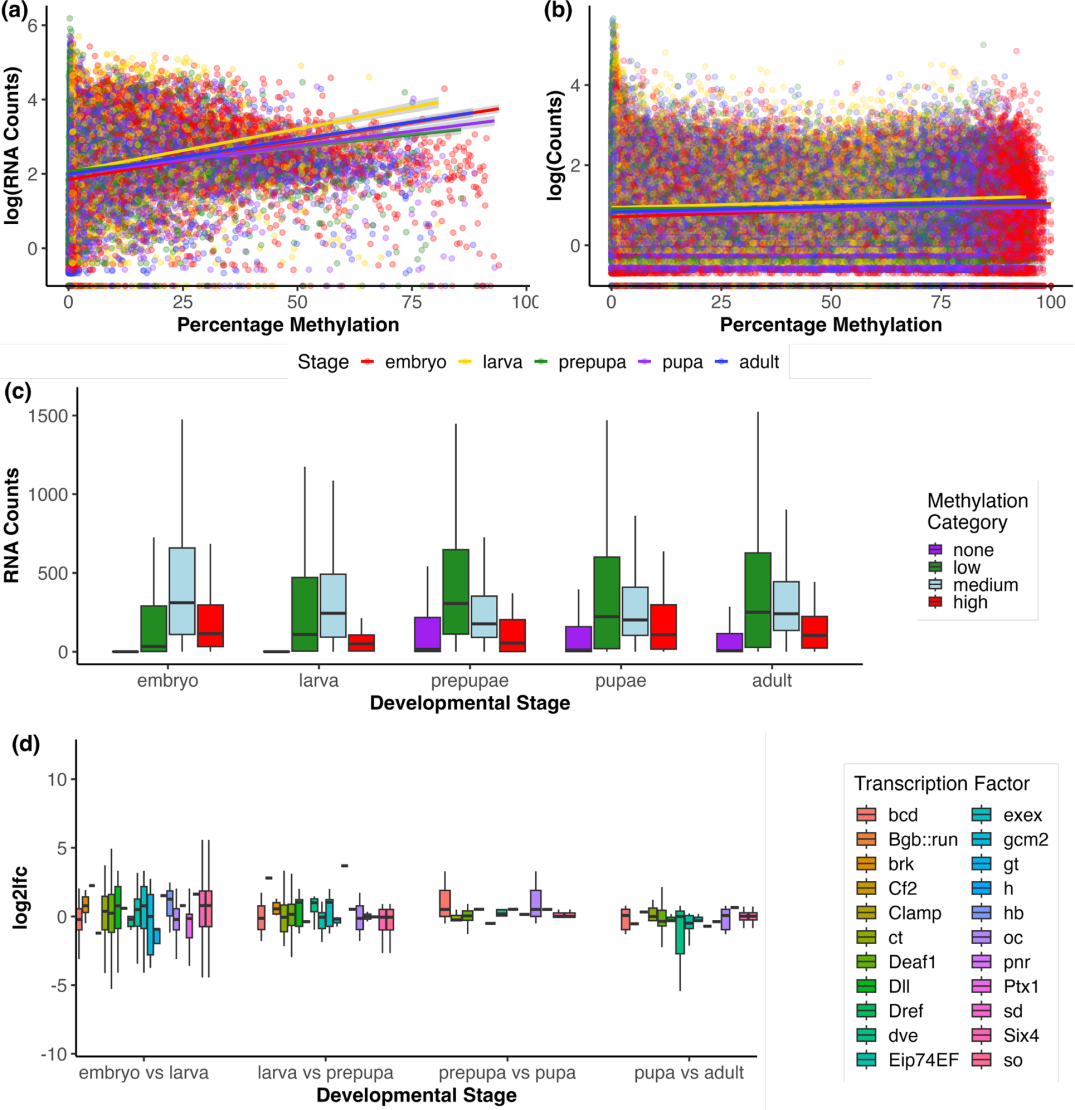
Linking DNA Methylation with Gene Expression. **A** Gene-level methylation versus gene RNA counts. **B** Exon-level methylation versus exon RNA counts. **C** Gene methylation category versus average RNA gene count. **D** Log fold changes in downstream genes of transcription factor binding sites that exhibit differential methylation. We plot these changes in each of the pairwise comparisons (embryo versus larva, larva versus prepupa, prepupa versus pupa and pupa versus adult). Boxes are omitted where the associated gene shows no change in expression; both significant and non-significant changes are included

To investigate whether DNA methylation influences gene expression, we employed multiple generalized linear models (GLMs). The first set of GLMs used a quasipoisson distribution to examine the relationship between gene-level methylation and gene expression (Table 1 row 1, Figure 4A), exon-level methylation and exon counts (Table 1 row 2, Figure 4B), gene methylation categories (none, low, medium, high) and gene expression (Table 1 row 3, Figure 4C), however, all models exhibited poor fit.

Next, we examined how differential methylation affected gene expression across developmental stages. Using Gaussian-distributed GLMs, we investigated the effect of the number of differentially methylated sites (DMS) within a gene on gene expression (Table 1 row 4), the influence of differentially methylated CpGs within transcription factor binding sites and their distance to the nearest transcription start site (TSS) (Table 1 row 5, Figure 4D), however both models had low pseudo-$R^2$ values, indicating weak predictive power. Of these genes downstream of differentally methylated transcription factor binding sites, 1609 / 1727 were differentially expressed.

**Table 1:** Summary of GLM results for DNA methylation and gene expression

Model	Chisq	df	p-value	Pseudo-$R^2$
**Quasipoisson distributed GLMs**				
Gene Counts $\sim$ Gene Meth. % $\times$ Stage	40.107	4	$4.1 \times 10^{-8}$	2.08
Exon Counts $\sim$ Exon Meth. % $\times$ Stage	9.9	4	0.0428	1.20
Gene Counts $\sim$ Meth. Category $\times$ Stage	39.8	12	$7.8 \times 10^{-5}$	2.30
**Gaussian distributed GLMs**				
LFC $\sim$ No. DMS $\times$ Pair Comp	100.72	3	$2.2 \times 10^{-16}$	2.1
LFC $\sim$ Distance TSS $\times$ Pairw Comp. $\times$ TF	11.27	27	1.00	5.3
LFC $\sim$ No. Consec. DMS $\times$ Pair Comp. $\times$ Distance TSS	0.083	2	0.96	26.0

Finally, we investigated whether the number of consecutive differentially methylated CpGs influenced the gene expression levels of the closest gene, as the study by Xu et al. [8] indicated that more methylated CpGs had a greater effect on gene expression. By selecting genomic locations that have two or more differentially methylated sites consecutively we were able to generate a model with better fit (26%, Table 1 row 6), but still not a good enough fit to make interpretations. Full tables of all these GLMs can be found in Supplementary Information4.

## Discussion

Our results demonstrate clear the developmental dynamics in DNA methylation across the life cycle of *Nasonia vitripennis*, emphasising stage-specific methylation that likely reflect epigenetic regulation of metamorphosis. Stage-specific methylation levels (1.1-$-$1.2% of CpGs) were stable, and within the range of previous *Nasonia* studies (0.63% [36] and 1.6% [17], with methylation predominantly localized to gene bodies, especially exons near the 5’ end (Figure 1B). We find that methylation is higher in exons than in introns, and that mean methylation peaks at exon 3, before decreasing as exon number increases (Supplementary Figure 1).

The majority of methylated CpGs were consistently methylated across developmental stages (Figure 1C), with motif analysis revealing homology binding sites of core biological processess including splicing regulation, chromatin structure, and gene regulation. The link between insect DNA methylation and splicing has long been proposed [37]. The fact that sites enriched for splicing regulators are amongst the most enriched of motifs that CpGs are located in across development further suggests the role of DNA methylation in insect splicing. However, exon usage did not correlate with exon methylation levels (Figure 4**C**). Furthermore, it is interesting to see that DNA methylated motifs across development being implicated in chromatin structure, given that the introduction of the de novo methyltransferase *Dnmt3a* results global hypermethylation and chromosomal defects in *Drosophila* [38].

### Global methylation dynamics across development

Methylation was highest in embryos and lowest in larvae (Figure 1A), coinciding with a large-scale demethylation event during the embryonic-larval transition (Figure 2B). Methylation levels rose again during the prepupal stage and stabilised in later stages, with local site-specific fluctuations. This pattern mirrors observations in the silk moth [7] and honeybees [39], although contrasts exist in other Hymenoptera, such as the carpenter ant *Camponotus floridanus* [40]. The presence of higher embryonic methylation in *Harpegnathos saltator* within the same study suggests that the carpenter ant may represent an evolutionary exception rather than a general trend among Hymenoptera.

### Stage-specific methylation mirrors metamorphosis

Throughout metamorphosis, we find indicators that DNA methylation plays a role in each stage of *Nasonia* development. For example, when performing GO term analysis of genes that possess CpGs uniquely methylated to each developmental stage, we find enrichment of stage-specific process. This is clear in the embryo where we find enrichment for gastrulation, and embryo morphogenesis, in the larva we find regionalization, anatomical structure morphogenesis, and epithelium development, and in the later stages of development we see action potential and epithelial cell development. The involvement of DNA methylation in gastrulation is unsurprising, given that *dnmt1a* knockouts lead to developmental arrest prior to gastrulation [3, 6]. Nor is the suggestion that methylation is involved in insect epithelial cell development surprising given previous suggestions in *Bombyx mori* that alterations in DNA methylation using the DNA methylation inhibitor (5-aza-dC) are implicated in chitin degradation [41].

When running motif analysis on the regions surrounding these CpGs which are uniquely methylated for each developmental stage, we find enrichment of proteins implicated in stage specific processes. For example, in the embryo we see enrichment for sites homologous to that of the segmentation transcription factor Slp and the mesodermal patterning factor Six4. In the prepupal stage, we find enrichment for sites homologous to the transcriptional repressor of fat body enhancers Aef1. In the pupal stage, we find enrichment for motifs homologous to binding sites of wing associated repressor Brk and transcription factor Nrf1. While the expression profile of some of these transcription factors (eg. Slp1, Pho and Six4) matched their methylation motif enrichment (Supplementary Figures2), others did not (eg. Vnd, Grh and Ewg (Nrf1), and the matching is likely a consequence of higher transcription factor expression in the embryo.

Analysis of genes containing differentially methylated CpGs revealed enrichment of GO terms appropriate for developmental transitions. For example, between the embryo and larva there were GO terms for gastrulation, larval development and embryo development, which pairs with previous findings linking insect DNA methylation with embryogenesis [3, 6] and larval development [41]. Between the larva and prepupa, we see enrichment for GO terms in morphogenesis and again larval development, whilst in the prepupa to pupal transition we also see the inclusion of epithelial cell development and signal transduction. In the pupal to adult transition, we see GO terms enriched for vasculature development, cellular response to stimulus, and again signal transduction.

### Dynamic expression of DNA methylation-related enzymes

*Tet* showed peak expression in embryos, aligning with demethylation during the embryonic-larval transition and suggesting a role in early developmental regulation. Therefore, the drop in methylation may be a consequence of tet’s demethylating role as highlighted by *Apis meliferra* Tet assays [42]. Tet functionality can be contradictory, as it can play a role in stabilizing methylation levels [43]. If such a scenario were true, decreases in methylation may instead be a consequence of replication-dependent passive loss of methylation [44]. Further studies are required to further elucidate the multifunctional roles of insect Tet enzymes, and how they regulate methylation dynamics through development. Conversely, *dnmt3* expression rose during the larval stage, consistent with a de novo methylation function. *Dnmt1c* expression was highest in the adult female, consistent with the thought that it may be an ovary specfic enzyme with a role in reproduction [18]. The high embryonic expression of *mbd* and *tip60* further supports the model of Xu et al. [8] that regulation of DNA methylation is particularly important early in development.

### Methylation-Associated regulation of gene expression

Despite observing developmental links between methylation and gene expression (Figures 4A–C), modeling methylation with transcriptomics yielded poor fits, reflecting the complexity of the influence of methylation. Genes with medium methylation tended to show higher expression in earlier stages, while lowly methylated genes were more highly expressed in later stages. This may reflect a developmental shift in methylation’s regulatory function.

When examining the expression profile of methylation associated genes and linking them with our methylation results, our findings follow the model proposed by Xu et al. [8], suggesting that in addition to recruiting Mbd and Tip60 to promote histone acetylation. The high embryonic expression of *mbd* suggests a prominent early role for Mbd-mediated regulation, with the drop in later stages possibly reflecting a transition to more flexible gene regulatory mechanisms. It should be noted that whilst *Bombyx mori mbd* possesses a canonical methyl-CpG-binding domain with high homology to mammals, Hymenoptera do not [45]. In fact, the exon possessing mCpG binding ability was lost early in Hymenoptera evolution. They instead possess an unique exon not found in other insect orders [45]. This additional exon is highly conserved amongst Hymenoptera, to the extent that this exon may even restore methylation binding ability [45], however biochemical analyses into Hymenoptera MBD methylation binding ability shows conflicting results [46, 47].

### Future directions

A key limitation in our attempts to correlate DNA methylation with transcriptomics is the reliance on whole-organism samples. The whole tissue results here highlight general trends of methylation across *Nasonia*’s wide range of cell types. However, to better understand precisely how methylation regulates gene expression across development tissue-specific or single-cell approaches should be applied, or alternatively the development of a *Nasonia* cell line. ChIP-seq for transcription factors whose motifs overlap with differentially methylated sites could clarify whether methylation modulates transcription factor occupancy. Furthermore, functional characterisation of *mbd* by RNA interference or CRISPR knockdowns would test its role in coordinating early developmental gene expression.

Another limitation to the study is that whole genome bisulphite sequencing can’t differentiate between 5mC methylation and 5mC hydroxymethylation. Previous studies have found enrichment of 5hmc in introns [48], which would lead one to expect a lower proportion of methylated CpG intronic sites (Figure 1B), and perhaps one would expect a higher proportion of 5hmC in the larval stage as 5hmC is an intermediate in the process of DNA demethylation. Future studies may instead wish to use techniques which would allow the identification of both 5mC and 5hmC.

An interesting conflict in our results is that most differential methylation occurs in the gene body, whilst transcription factors traditionally bind in the promoter region or at enhancers. In mammals, intrageneic CpG islands have been linked to morphogenesis and cell lineage [49], suggesting DNA methylation plays a role in regulating differentiation. We may be seeing a similar role of gene body methylation regulating differentiation in *Nasonia*. Future work utilising dCas9-DNMT fusions could verify these links, by investigating if increasing gene body methylation increases gene expression.

## Conclusion

This study provides the first map of DNA methylation dynamics throughout development in *Nasonia vitripennis*. We identified global fluctuations in methylation levels, with peak levels in embryos and significant demethylation by the larval stage, followed by reprogramming during metamorphosis. Stage-specific methylation changes were associated with developmental transcription factor motifs, suggesting a regulatory role in metamorphosis. Although stage-specific methylation paired with stage-specific gene expression transitions, the underlying mechanisms are complex and warrant further investigation. These findings highlight the importance of DNA methylation in regulating developmental transitions and outline future directions for functional studies on epigenetic regulation in insects.

## Supplementary information

Supplementary material 1.

Supplementary material 2. Results from DNA methylation analyses

Supplementary material 3. Results from RNA analyses

Supplementary material 4. Statistical results from GLMs linking DNA methylation to gene expression.

## Data Availability

Whole genome bisulfite sequencing files are available on EBI under the accession number E-MTAB-14656. RNA libraries are publicly available on EBI under the accession number E-MTAB-14657.

## References

[1] Hajkova P, Erhardt S, Lane N, Haaf T, El-Maarri O, Reik W, et al. Epigenetic reprogramming in mouse primordial germ cells. Mech Dev. 2002;117(1):15–23. 10.1016/S0925-4773(02)00181-8. 12204247 10.1016/s0925-4773(02)00181-8

[2] Mayer W, Niveleau A, Walter J, Fundele R, Haaf T. Demethylation of the zygotic paternal genome. Nature. 2000;403(6769):501–2. 10.1038/35000656. 10676950 10.1038/35000656

[3] Zwier MV, Verhulst EC, Zwahlen RD, Beukeboom LW, Zande L. DNA methylation plays a crucial role during early Nasonia development. Insect Mol Biol. 2012;21(1):129–38. 10.1111/j.1365-2583.2011.01121.x. 22122805 10.1111/j.1365-2583.2011.01121.x

[4] Bewick AJ, Sanchez Z, Mckinney EC, Moore AJ, Moore PJ, Schmitz RJ. Dnmt1 is essential for egg production and embryo viability in the large milkweed bug. Oncopeltus Fasciatus Epigenetics & Chromatin. 2019;12(1):6. 10.1186/s13072-018-0246-5. 30616649 10.1186/s13072-018-0246-5 PMC6322253

[5] Ventós-Alfonso A, Ylla G, Montañes J-C, Belles X. DNMT1 promotes genome methylation and early embryo development in cockroaches. iScience. 2020;23(12):101778. 10.1016/j.isci.2020.101778. 33294787 10.1016/j.isci.2020.101778 PMC7691181

[6] Arsala D, Wu X, Yi SV, Lynch JA. Dnmt1a is essential for gene body methylation and the regulation of the zygotic genome in a wasp. PLoS Genet. 2022;18(5):1010181. 10.1371/journal.pgen.1010181. 10.1371/journal.pgen.1010181 PMC9075658 35522715

[7] Xu G-F, Gong C-C, Tian Y-L, Fu T-Y, Lin Y-G, Lyu H, et al. DNA methylation-mediated expression of zinc finger protein 615 affects embryonic development in bombyx mori. Zool Res. 2022;43(4):552–65. 10.24272/j.issn.2095-8137.2022.031. 35616260 10.24272/j.issn.2095-8137.2022.031 PMC9336445

[8] Xu G, Lyu H, Yi Y, Peng Y, Feng Q, Song Q, et al. Intragenic DNA methylation regulates insect gene expression and reproduction through the MBD/Tip60 complex. iScience. 2021;24(2):102040. 10.1016/j.isci.2021.102040. 33521602 10.1016/j.isci.2021.102040 PMC7820559

[9] Deshmukh S, Ponnaluri VC, Dai N, Pradhan S, Deobagkar D. Levels of DNA cytosine methylation in the drosophila genome. PeerJ. 2018;6:5119. 10.7717/peerj.5119. 10.7717/peerj.5119 PMC6033079 30002967

[10] Zhang T, Song W, Li Z, Qian W, Wei L, Yang Y, et al. Krüppel homolog 1 represses insect ecdysone biosynthesis by directly inhibiting the transcription of steroidogenic enzymes. Proc Natl Acad Sci. 2018;115(15):3960–5. 10.1073/pnas.1800435115. 29567866 10.1073/pnas.1800435115 PMC5899488

[11] Du Q, Luu P-L, Stirzaker C, Clark SJ. Methyl-CpG-binding domain proteins: readers of the epigenome. Epigenomics. 2015;7(6):1051–73. 10.2217/epi.15.39. 25927341 10.2217/epi.15.39

[12] Héberlé Î, Bardet A. Sensitivity of transcription factors to DNA methylation. Essays Biochem. 2019;63(6):727–41. 10.1042/EBC20190033. 31755929 10.1042/EBC20190033 PMC6923324

[13] Wang X, Werren JH, Clark AG. Allele-specific transcriptome and methylome analysis reveals stable inheritance and cis-regulation of DNA methylation in Nasonia. PLoS Biol. 2016;14(7):1002500. 10.1371/journal.pbio.1002500. 10.1371/journal.pbio.1002500 PMC4933354 27380029

[14] Werren JH, Richards S, Desjardins CA, Niehuis O, Gadau J, Colbourne JK, et al. Functional and evolutionary insights from the genomes of three parasitoid Nasonia species. Science. 2010;327(5963):343–8. 10.1126/science.1178028. 20075255 10.1126/science.1178028 PMC2849982

[15] Brink K, Thomas CL, Jones A, Chan TW, Mallon EB. Exploring the ageing methylome in the model insect. Nasonia vitripennis BMC Genom. 2024;25(1):305. 10.1186/s12864-024-10211-7. 10.1186/s12864-024-10211-7 PMC10958858 38519892

[16] Werren JH, Loehlin DW. The parasitoid wasp Nasonia: an emerging model system with haploid male genetics. Cold Spring Harb Protoc. 2009;2009(10):134. 10.1101/pdb.emo134. 10.1101/pdb.emo134 PMC2916733 20147035

[17] Wang X, Wheeler D, Avery A, Rago A, Choi J-H, Colbourne JK, et al. Function and evolution of DNA methylation in Nasonia vitripennis. PLoS Genet. 2013;9(10):1003872. 10.1371/journal.pgen.1003872. 10.1371/journal.pgen.1003872 PMC3794928 24130511

[18] Thomas CL. Nasonia vitripennis: an insect model for DNA methylation. PhD Thesis, University of Leicester, 2023. https://figshare.le.ac.uk/articles/thesis/Nasonia_vitripennis_An_Insect_Model_for_DNA_Methylation/21960113 Accessed 19 Aug 2024

[19] Dalla Benetta E, Antoshechkin I, Yang T, Nguyen HQM, Ferree PM, Akbari OS. Genome elimination mediated by gene expression from a selfish chromosome. Sci Adv. 2020;6(14):9808. 10.1126/sciadv.aaz9808. 10.1126/sciadv.aaz9808 PMC7124933 32284986

[20] Krueger F, Andrews SR. Bismark: a flexible aligner and methylation caller for bisulfite-seq applications. Bioinformatics. 2011;27(11):1571–2. 10.1093/bioinformatics/btr167. 21493656 10.1093/bioinformatics/btr167 PMC3102221

[21] Akalin A, Kormaksson M, Li S, Garrett-Bakelman FE, Figueroa ME, Melnick A, et al. methylKit: a comprehensive r package for the analysis of genome-wide DNA methylation profiles. Genome Biol. 2012;13(10):87. 10.1186/gb-2012-13-10-r87. 10.1186/gb-2012-13-10-r87 PMC3491415 23034086

[22] Dainat J. NBISweden/AGAT: AGAT-v1.4.0. Zenodo. 2024. https://zenodo.org/records/11106497. Accessed 19 Aug 2024

[23] Tan G, Lenhard B. TFBSTools: an R/bioconductor package for transcription factor binding site analysis. Bioinformatics. 2016;32(10):1555–6. 10.1093/bioinformatics/btw024. 26794315 10.1093/bioinformatics/btw024 PMC4866524

[24] Rauluseviciute I, Riudavets-Puig R, Blanc-Mathieu R, Castro-Mondragon J, Ferenc K, Kumar V, et al. JASPAR 2024: 20th anniversary of the open-access database of transcription factor binding profiles. Nucleic Acids Res. 2024;52(D1):174–82. 10.1093/nar/gkad1059. 10.1093/nar/gkad1059 PMC10767809 37962376

[25] Heinz S, Benner C, Spann N, Bertolino E, Lin YC, Laslo P, et al. Simple combinations of lineage-determining transcription factors prime cis-regulatory elements required for macrophage and B cell identities. Mol Cell. 2010;38(4):576–89. 10.1016/j.molcel.2010.05.004. 20513432 10.1016/j.molcel.2010.05.004 PMC2898526

[26] Cantalapiedra CP, Hernández-Plaza A, Letunic I, Bork P, Huerta-Cepas J. eggNOG-mapper v2: functional annotation, orthology assignments, and domain prediction at the metagenomic scale. Mol Biol Evol. 2021;38(12):5825–9. 10.1093/molbev/msab293. 34597405 10.1093/molbev/msab293 PMC8662613

[27] Alexa A, Rahnenfuhrer J. Gene set enrichment analysis with topGO

[28] Yu G, Wang L-G, Han Y, He Q-Y. ClusterProfiler: an R package for comparing biological themes among gene clusters. OMICS. 2012;16(5):284–7. 10.1089/omi.2011.0118. 22455463 10.1089/omi.2011.0118 PMC3339379

[29] Dobin A, Davis CA, Schlesinger F, Drenkow J, Zaleski C, Jha S, et al. STAR: ultrafast universal RNA-seq aligner. Bioinformatics. 2013;29(1):15–21. 10.1093/bioinformatics/bts635. 23104886 10.1093/bioinformatics/bts635 PMC3530905

[30] Anders S, Pyl PT, Huber W. HTSeq-a python framework to work with high-throughput sequencing data. Bioinformatics. 2015;31(2):166–9. 10.1093/bioinformatics/btu638. 25260700 10.1093/bioinformatics/btu638 PMC4287950

[31] Love MI, Huber W, Anders S. Moderated estimation of fold change and dispersion for RNA-seq data with DESeq2. Genome Biol. 2014;15(12):550. 10.1186/s13059-014-0550-8. 25516281 10.1186/s13059-014-0550-8 PMC4302049

[32] Wickham H. Ggplot2. WIREs Comput Stat. 2011;3(2):180–5. 10.1002/wics.147.

[33] Li Y, Rao X, Mattox WW, Amos CI, Liu B. RNA-seq analysis of differential splice junction usage and intron retentions by DEXSeq. PLoS ONE. 2015;10(9):0136653. 10.1371/journal.pone.0136653. 10.1371/journal.pone.0136653 PMC4556662 26327458

[34] Bray NL, Pimentel H, Melsted P, Pachter L. Near-optimal probabilistic RNA-seq quantification. Nat Biotechnol. 2016;34(5):525–7. 10.1038/nbt.3519. 27043002 10.1038/nbt.3519

[35] Hunt BJ, Pegoraro M, Marshall H, Mallon EB. A role for DNA methylation in bumblebee morphogenesis hints at female-specific developmental erasure. Insect Mol Biol. 2024;33(5):481–92. 10.1111/imb.12897. 38348493 10.1111/imb.12897

[36] Beeler SM, Wong GT, Zheng JM, Bush EC, Remnant EJ, Oldroyd BP, et al. Whole-genome DNA methylation profile of the jewel wasp (Nasonia vitripennis). G3 (Bethesda, Md). 2014;4(3):383–8. 10.1534/g3.113.008953. 24381191 10.1534/g3.113.008953 PMC3962478

[37] Li-Byarlay H, Li Y, Stroud H, Feng S, Newman TC, Kaneda M, et al. RNA interference knockdown of DNA methyl-transferase 3 affects gene alternative splicing in the honey bee. Proc Natl Acad Sci. 2013;110(31):12750–5. 10.1073/pnas.1310735110. 23852726 10.1073/pnas.1310735110 PMC3732956

[38] Weissmann F, Muyrers-Chen I, Musch T, Stach D, Wiessler M, Paro R, et al. DNA hypermethylation in drosophila melanogaster causes irregular chromosome condensation and dysregulation of epigenetic histone modifications. Mol Cell Biol. 2003;23(7):2577–86. 10.1128/MCB.23.7.2577-2586.2003. 12640138 10.1128/MCB.23.7.2577-2586.2003 PMC150732

[39] Harris KD, Lloyd JPB, Domb K, Zilberman D, Zemach A. DNA methylation is maintained with high fidelity in the honey bee germline and exhibits global non-functional fluctuations during somatic development. Epigenetics & Chromatin. 2019;12(1):62. 10.1186/s13072-019-0307-4. 31601251 10.1186/s13072-019-0307-4 PMC6786280

[40] Bonasio R, Li Q, Lian J, Mutti NS, Jin L, Zhao H, et al. Genome-wide and caste-specific DNA methylomes of the ants camponotus floridanus and harpegnathos saltator. Current biology CB. 2012;22(19):1755–64. 10.1016/j.cub.2012.07.042. 22885060 10.1016/j.cub.2012.07.042 PMC3498763

[41] Xu G, Yi Y, Lyu H, Gong C, Feng Q, Song Q, et al. DNA methylation suppresses chitin degradation and promotes the wing development by inhibiting bmara-mediated chitinase expression in the silkworm. Bombyx mori Epigenetics & Chromatin. 2020;13:34. 10.1186/s13072-020-00356-6. 32887667 10.1186/s13072-020-00356-6 PMC7472703

[42] Wojciechowski M, Rafalski D, Kucharski R, Misztal K, Maleszka J, Bochtler M, et al. Insights into DNA hydroxymethylation in the honeybee from in-depth analyses of tet dioxygenase. Open Biol. 2014;4(1):140110. 10.1098/rsob.140110. 25100549 10.1098/rsob.140110 PMC4150289

[43] Santiago M, Antunes C, Guedes M, Iacovino M, Kyba M, Reik W, et al. Tet3 regulates cellular identity and DNA methylation in neural progenitor cells. Cell Mol Life Sci CMLS. 2020;77(14):2871–83. 10.1007/s00018-019-03335-7. 31646359 10.1007/s00018-019-03335-7 PMC7326798

[44] Wu H, Zhang Y. Reversing DNA methylation: mechanisms, genomics, and biological functions. Cell. 2014;156:45–68. 10.1016/j.cell.2013.12.019. 24439369 10.1016/j.cell.2013.12.019 PMC3938284

[45] Israel E, Länger ZM, Heckenhauer J, Kurtz J, Prohaska S. MBD2/3 lost its methyl-CpG binding ability in multiple families of Holometabola. bioRxiv. Pages: 2025.04.22.650097 Section: New Results. 2025. 10.1101/2025.04.22.650097.

[46] Wang Y, Jorda M, Jones PL, Maleszka R, Ling X, Robertson HM, et al. Functional CpG methylation system in a social insect. Science. 2006;314(5799):645–7. 10.1126/science.1135213. 17068262 10.1126/science.1135213

[47] Liu K, Lei M, Wu Z, Gan B, Cheng H, Li Y, et al. Structural analyses reveal that MBD3 is a methylated CG binder. FEBS J. 2019;286(16):3240–54. 10.1111/febs.14850. 30980593 10.1111/febs.14850

[48] Cingolani P, Cao X, Khetani RS, Chen C-C, Coon M, Sammak A, et al. Intronic Non-CG DNA hydroxymethylation and alternative mRNA splicing in honey bees. BMC Genom. 2013;14(1):666. 10.1186/1471-2164-14-666. 10.1186/1471-2164-14-666 PMC3850688 24079845

[49] Lee S-M, Lee J, Noh K-M, Choi W-Y, Jeon S, Oh GT, et al. Intragenic CpG islands play important roles in bivalent chromatin assembly of developmental genes. Proc Natl Acad Sci USA. 2017;114(10):1885–94. 10.1073/pnas.1613300114. 10.1073/pnas.1613300114 PMC5347632 28223506

