# Supplementary material for: Genome-wide demethylation and targeted remethylation during metamorphosis in the jewel wasp Nasonia vitripennis
Source: Epigenetics Chromatin. 2025 Dec 4;18:79. doi: 10.1186/s13072-025-00639-w (PMC12696957; doi:10.1186/s13072-025-00639-w)
Supplement: Supplementary file 1 — Supplementary material 1. [file 13072_2025_639_MOESM1_ESM.pdf]

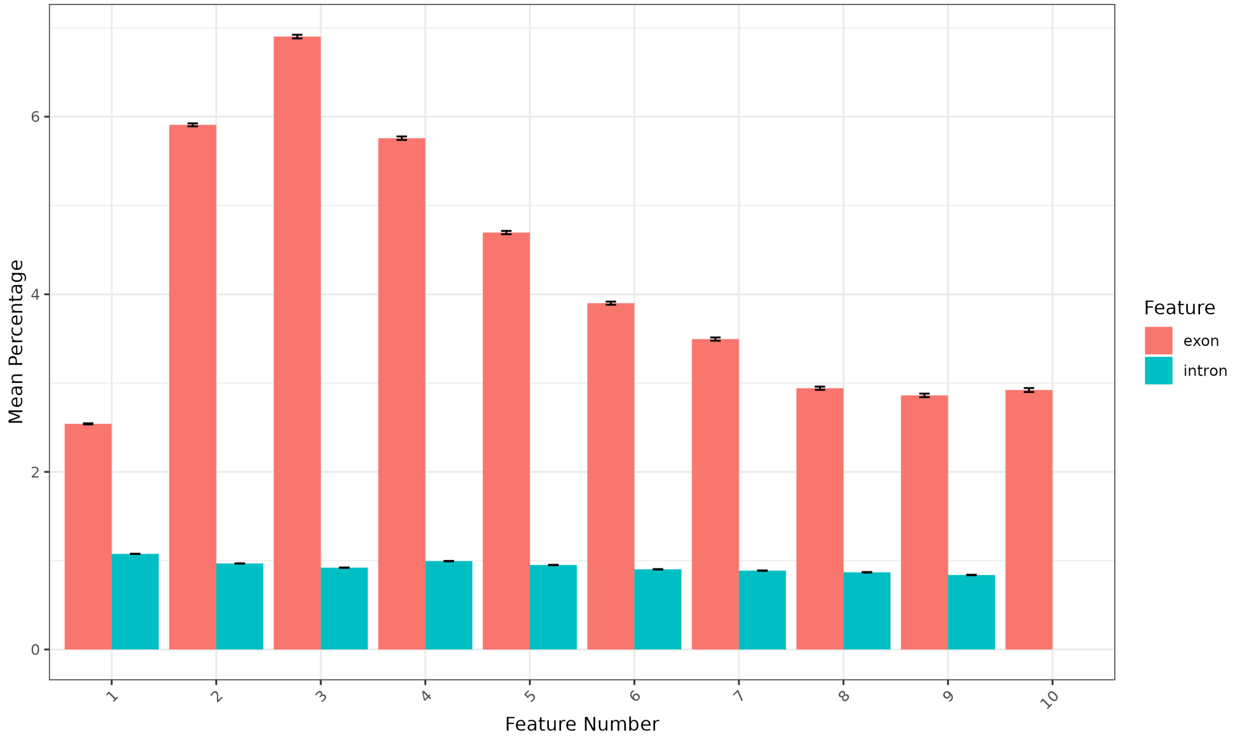

Supplementary Figure 1

Mean Percentage CpG Methylation (of methylated sites) per Exon/Intron (with x-axis donating exon / intron number

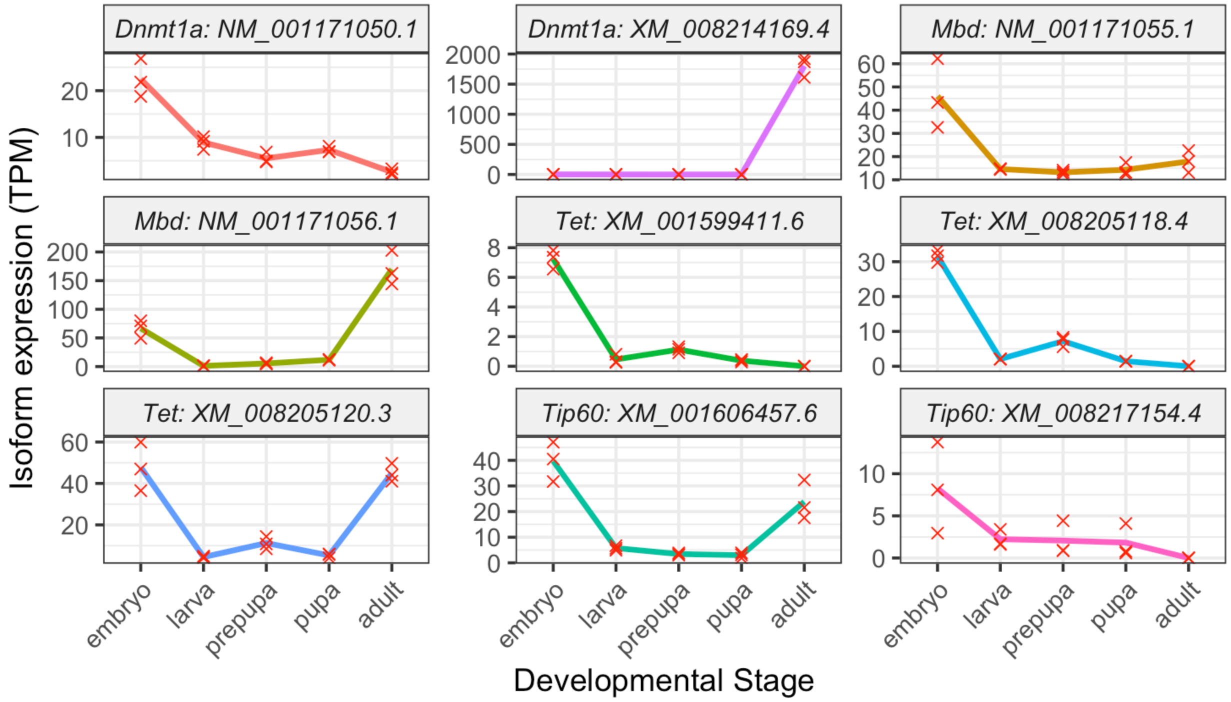

Supplementary Figure 2

Isoform TPM for Methylation associated genes. Where omitted, methylation associated genes only had one isoform.

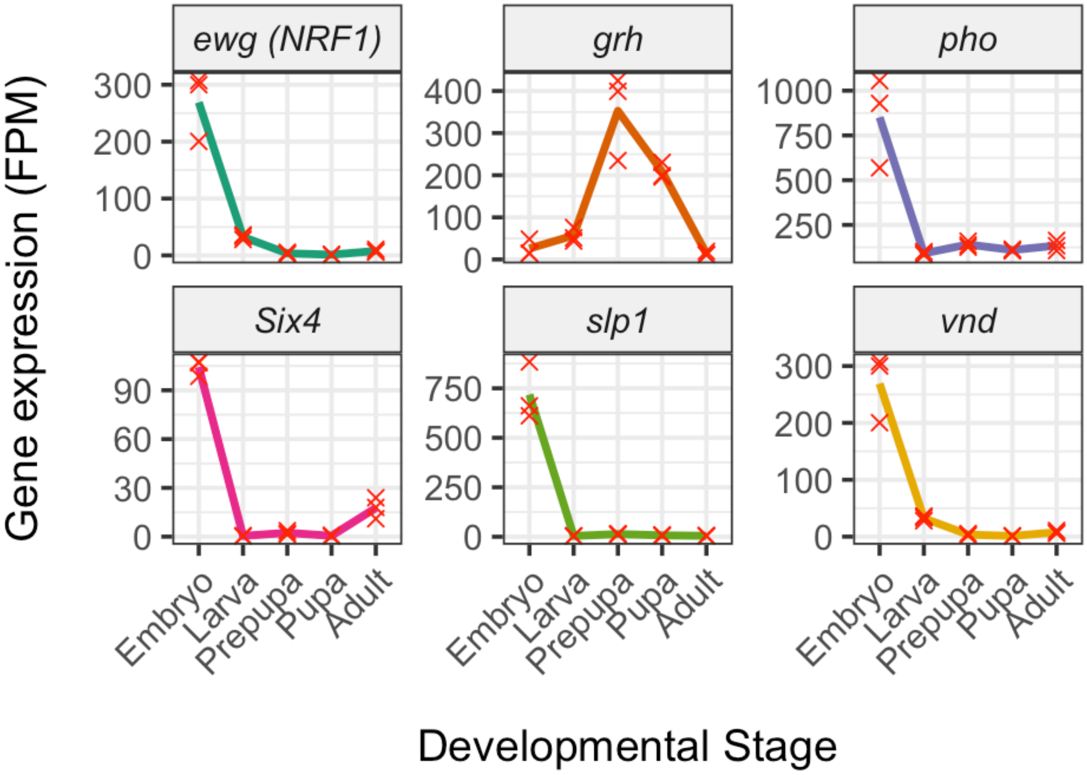

Supplementary Figure 3

Gene Expression counts for transcription factors associated with DNA methylation binding.
